# Microporous structures on mineralized collagen mediate bone restoration by promoting nucleolin secretion to induce macrophage M2 polarization

**DOI:** 10.1093/rb/rbaf079

**Published:** 2025-09-04

**Authors:** Jun Li, Yujiao Liu, Chunxiu Meng, Yujue Zhang, Yurun Luan, Kun Liu, Lina Zhang, Fengzhen Liu, Xin Luo, Bin Zhang

**Affiliations:** Liaocheng People’s Hospital, Liaocheng Hospital Affiliated Shandong First Medical University, Liaocheng 252000, China; Department of Oral and Maxillofacial Surgery, School and Hospital of Stomatology, Shandong University & Shandong Provincial Key Laboratory of Oral Tissue Regeneration & Shandong Engineering Laboratory for Dental Materials and Oral Tissue Regeneration, Jinan, Shandong, 250100, China; Liaocheng People’s Hospital, Liaocheng Hospital Affiliated Shandong First Medical University, Liaocheng 252000, China; Liaocheng People’s Hospital, Liaocheng Hospital Affiliated Shandong First Medical University, Liaocheng 252000, China; Liaocheng People’s Hospital, Liaocheng Hospital Affiliated Shandong First Medical University, Liaocheng 252000, China; Liaocheng People’s Hospital, Liaocheng Hospital Affiliated Shandong First Medical University, Liaocheng 252000, China; Liaocheng People’s Hospital, Liaocheng Hospital Affiliated Shandong First Medical University, Liaocheng 252000, China; Liaocheng People’s Hospital, Liaocheng Hospital Affiliated Shandong First Medical University, Liaocheng 252000, China; Liaocheng People’s Hospital, Liaocheng Hospital Affiliated Shandong First Medical University, Liaocheng 252000, China; Liaocheng People’s Hospital, Liaocheng Hospital Affiliated Shandong First Medical University, Liaocheng 252000, China; Liaocheng People’s Hospital, Liaocheng Hospital Affiliated Shandong First Medical University, Liaocheng 252000, China

**Keywords:** mineralized collagen, microporous structures, macrophage polarization, NCL expression, bone restoration

## Abstract

Bone defects rehabilitation is one of the difficulties in oral clinical practice. Implanted biomaterials have pivotal effects on the regeneration in critical bone defects, but the immunologic reactions arising from their entering into the body are difficult to control. Biomaterials characteristics can effect the immune response, and thus, interfere with the skeletal system. Our previous study found that microporous structures on mineralized collagen (MC) modulated macrophage polarization in bone immune response thereby promoting osteogenic differentiation of osteoblasts. However, the role of MC with various microporous structures in guiding bone rejuvenation *in vivo* is still unknown and the specific mechanism of crosstalk between MC, macrophages and osteoblasts during bone repair is poorly understood. In this research, we investigated the impact and mechanism of MC with different pore sizes on bone regeneration. The results showed that MC with a medium pore size (85 μm) promoted bone defects repair significantly, M2 macrophage polarization and nucleolin (NCL) expression in macrophages. And the fanconi anemia pathway was implicated in this process. We found that NCL regulated macrophage polarization towards M2 by inhibiting and overexpressing NCL in macrophages. This study will provide a new idea for using biomaterials to regulate host immune response and promote bone regeneration.

## Graphical abstract

**Figure:**
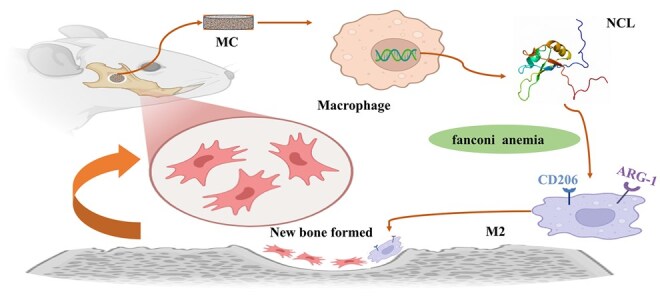

## Introduction

The repair and reconstruction of jaw defects caused by trauma, inflammation, tumor and other reasons is the difficulty of oral and maxillofacial surgery [1]. Currently, the main treatment methods are autologous bone transplantation, allogeneic bone transplantation and distraction osteogenesis. Among them, autologous bone transplantation has the disadvantages of insufficient bone mass and functional damage of donor site. There is immune rejection of allogeneic bone, and the longer course of distraction osteogenesis often leads to complications such as muscle atrophy and infection [2, 3]. Bone repair materials have emerged as an alternative to bone tissue regeneration [4].

As a foreign body, the bone repair material triggers a host immune response after implantation. Excessive immune response may eventually lead to implantation failure, which largely limits the applications of biomaterials. Traditional design focuses on the interaction between bone repair materials and osteoblasts, and the principle has been to minimize the immune response by preparing inert materials. With the intensive research in osteoimmunology, the effect of immune response in the process of material induced osteogenesis has been increasingly emphasized [5]. Meng *et al*. found that mineralised collagen (MC) stimulated macrophages to secrete adrenomedullin, creating an ideal bone immune microenvironment for bone defect repair [6]. Thus, an appropriate immune response facilitated the integration and functional stability of biomaterials. The design and development of biomaterials has shifted from “immune evasion” to “immune modulation” which improves tissue healing and regeneration by modulating the inflammatory response [7].

The inflammatory response is critical to bone healing. The suppression of acute inflammation results in poor bone repair. However, excessive acute inflammation and persistent chronic inflammation resulting from severe injury are detrimental to bone healing [8]. In the acute inflammatory stage, macrophages phagocytose invading microorganisms and tissue debris to maintain tissue homeostasis and release pro-inflammatory cytokines. In the middle and late stages of the repair response, macrophages reduce the inflammatory response of the tissue by secreting anti-inflammatory cytokines. The macrophage phenotype that promotes inflammation is known as M1 macrophage, and the promotion of tissue repair is denoted as M2 macrophage. The change in function and phenotype is described to be macrophage polarization [9, 10]. The change and homeostasis of macrophage phenotype is important for bone regeneration. Chen *et al*. showed that lysine-specific demethylase 6B plays an active role in osteogenic differentiation by reducing the ratio of M1/M2 [11].

MC is composed of ordered arrangement of type I collagen and nano-hydroxyapatite [12]. Our previous studies found that MC with different microporous structures affected osteoblast differentiation by influencing macrophage polarization [13]. However, the effect of MC microporous structure on the repair of jaw defects is still unclear, and the specific mechanism of crosstalk between macrophages and osteoblasts has not been completely clarified. NCL is a multifunctional protein involved in a variety of cellular life activities and consists of three separate structural domains that can bind DNA, RNA and proteins, respectively, to fulfill their corresponding functions [14]. In this investigation, we demonstrated that MC with a pore size of 85 μm promoted the repair of rat jaw defects by promoting macrophage M2 polarization, in which NCL played a critical function. In depth study of the immune reaction after implantation of biomaterials and exploring the cells and factors that play a key role in it will help to design and develop biomaterials with immunomodulatory properties, so as to better guide the tissue response to achieve the function of biomaterials.

## Materials and methods

### Preparation of MC scaffolds with different pore sizes

MC is prepared as described previously [13]. The process employed purified and deantigenized type I collagen (0.67 g/L) as a structural template for biomimetic mineralization within a calcium-phosphate solution. Precise quantities of CaCl_2_ and H_3_PO_4_ solutions were combined with the collagen, maintaining a Ca/P molar ratio of 1.67. Under gentle stirring at ambient temperature, the mixture’s pH was stabilized at 7.4 using sodium hydroxide. Following a 48-hr reaction period, the resulting precipitate underwent washing, filtration and comprehensive lyophilization to yield the final porous MC material. Three distinct scaffold variants (MC-A, MC-B, MC-C) were engineered, differing primarily in pore architecture. MC-A features large pores with an average diameter of 273 ± 13 μm and high porosity (80–90%); MC-B has medium pores averaging 84 ± 3 μm in diameter with moderate porosity (70–80%); and MC-C possesses small pores averaging 9.7 ± 0.2 μm in diameter with lower porosity (50–60%).

### Cell culture

The macrophage cell line RAW264.7 was seeded on the surface of three groups of MC scaffolds in 6-well plates at a density of 1 × 10^6^ cells per well. The cells were incubated with complete DMEM medium (10% fetal bovine serum + 1% penicillin/streptomycin).The cells on the surface of the material were digested with trypsin. After centrifugation at 1000 rpm for 5 min and 1 ml TRIzol Reagent (Takara, Japan) was added to re-suspend the cells. Total RNA extraction and transcriptome sequencing were conducted by Beijing Novogene Technology Co.

### Transcriptome sequencing

RNA integrity was assessed using the RNA Nano 6000 Assay Kit of the Bioanalyzer 2100 system. Total RNA served as the starting material for RNA library preparation. Briefly, mRNA was isolated from total RNA using poly-T oligo-conjugated magnetic beads. Fragmentation was performed with divalent cations at elevated temperature in First Strand Synthesis Reaction Buffer (5X). First-strand cDNA synthesis utilized random hexamer primers and M-MuLV Reverse Transcriptase. RNA templates were subsequently degraded with RNase H. Second-strand cDNA was generated using DNA polymerase I and dNTPs. Exonuclease/polymerase activities then converted any remaining overhangs into blunt ends. Following adenylation of DNA fragment 3' ends, adaptors featuring a hairpin loop structure were ligated. To enrich for cDNA fragments within the 3 7 0–420 bp size range, library fragments underwent purification using the AMPure XP system (Beckman Coulter, Beverly, USA). PCR amplification was conducted, and the resulting product was purified again with AMPure XP beads to yield the final library. Library quality control involved initial quantification via Qubit 2.0 Fluorometer, dilution to 1.5 ng/µl, and assessment of insert size distribution on an Agilent 2100 bioanalyzer. Libraries meeting the expected insert size criteria were then precisely quantified via qRT-PCR to determine effective concentration (requiring >2 nM). Qualified libraries were pooled based on effective concentration and desired sequencing depth prior to sequencing on the Illumina NovaSeq 6000 platform.

### Differential expression genes analysis

Differential expressed genes (DEGs) analysis of different groups was performed using the DESeq2 R package (1.20.0). Padj <= 0.05 and |log2(foldchange)| >= 1 were set as the threshold for significantly differential expression.

### Enrichment analysis of DEGs

Gene Ontology (GO) enrichment analysis of DEGs was implemented by the clusterProfiler R package. The clusterProfiler R package was employed to test the statistical enrichment of DEGs in KEGG pathways.

### qRT-PCR

Total RNA was isolated from cells using TRIzol Reagent (Takara, Japan). According to the manufacturer’s instructions, the PrimeScript RT Reagent Kit (Takara, Japan) was applied for reverse transcription. TB GREEN Premix EX Taq (Takara, Japan) was performed for qRT-PCR. Primer sequences are shown in Table 1.

**Table 1.** Primer sequence

Gene	Primer	Sequence (5′–3′)
*NCL*	Forward	GGGCAGAACAATCAGGCT
	Reverse	CACCTCTGCCTCCGAAAC
*ARG-1*	Forward	CTCCAAGCCAAAGTCCTTAGAG
	Reverse	AGGAGCTGTCATTAGGGACATC
*iNOS*	Forward	CACCAAGCTGAACTTGAGCG
	Reverse	CACCAAGCTGAACTTGAGCG
*GAPDH*	Forward	TGACCACAGTCCATGCCATC
	Reverse	GACGGACACATTGGGGGTAG

### Cell counting kit-8

AS1411 was produced by Shanghai Shenggong Biological Co., Ltd, with the sequence of 5′-T-(C6-S-S-C6)-TTGGTGGTGGTGGTTGTGGTGGTGGTGG-3′. RAW264.7 cells were seeded in 96-well plates with 3000–5000 cells per well. The cells were cultured with complete medium containing different concentrations of AS1411 (0 μM, 2.5 μM, 5 μM, 10 μM) on the second day. Three duplicate wells per group. 100 μL culture medium containing 10% cell counting kit-8 (CCK-8) solution was added to each well and incubated for 2 hr at 1 day, 2 days and 3 days, respectively. The absorbance value of each group at 450 nm wavelength was detected by microplate reader.

### Western blot

The cells lysed on ice for 40 min were centrifuged at 13000 r/min for 15 min, and the supernatant was retained. Protein concentration was determined by enhanced BCA protein assay kit (Beyotime, China). The SDS-PAGE sample loading buffer (Beyotime, China) was added at a volume ratio of 4:1 and denatured at 100°C for 10 min. Total protein was separated by 10–15% SDS-PAGE. The total protein was separated by SDS-PAGE and transferred to PVDF membrane. The membrane was blocked with 5% skim milk and incubated with NCL, ARG-1, iNOS and GAPDH primary antibodies at a dilution ratio of 1:1000 at 4°C overnight. After washing, the membrane was incubated with secondary antibody. Finally, the bands were detected by BeyoECL Plus (Beyotime, China). Grayscale analysis was performed via Image J software.

### Lentivirus transfection

RAW264.7 cells were prepared into a cell suspension with a density of 1 × 10^5^ cells/mL, and 2 mL was added to a 6-well plate. The cells were infected with the culture medium containing lentivirus (LV-NCL) without FBS the next day. The complete medium was replaced after 12 hr. The cells were cultured for 24 h and 2 μg/mL puromycin was added. The RAW264.7 cell line overexpressing NCL was constructed after 48 hr of screening. The expression of green fluorescent protein was observed under an inverted fluorescence microscope at 80% of the cells. Macrophages transfected with Lv-NC and Lv-NCL lentiviruses were seeded into 6-well plates at 1 × 10^6^ cells per well. Total RNA and proteins were extracted after 3 days to detect the expression of NCL, iNOS (M1 marker) and ARG-1 (M2 marker).

### Animal surgery

All animal investigations were approved by the Ethics Committee of Liaocheng People 's Hospital (D2022026). The 8-week-old SD male rats were anesthetized by intraperitoneal injection of 2% pentobarbital sodium. Under general anesthesia, an incision on the skin along the inferior margin of left mandible was performed. The soft tissue was separated and the surface of bone was exposed. Bone defects with a diameter of 4 mm were prepared with a circular drill and implanted into MC-A, MC-B and MC-C, respectively. Then the muscle and skin layers were sutured. The control group was directly sutured after bone defect preparation. Antibiotics were given for 3 days after operation. The mandibular specimens were fixed in paraformaldehyde for 24 h at 1 week, 4 weeks and 8 weeks after operation. After X-ray scanning (63 kV, 8 mA, 0.16 s), they were decalcified in 10% EDTA solution for 8 weeks.

### Histological staining

The jaw bone tissue was decalcified and embedded in paraffin, and 5 μm thick sections were cut. The sections were dewaxed and hydrated in various volume fractions of ethanol, followed by staining with hematoxylin and eosin (HE), and Masson’s trichrome to analyze the tissue morphology.

### IHC staining

Paraffin sections were blocked endogenous peroxidase by 3% H_2_O_2_. After antigen retrieval, CD86, iNOS, ARG-1, NCL and OCN primary antibodies (1:200) were incubated overnight at 4°C. The sections were incubated with HRP-labeled secondary antibody at 37°C for 1 hr, and DAB was added dropwise. Image-Pro Plus software measured the integrated option density (IOD) and calculated the mean option density (MOD) of the bone defect area after taking pictures with a microscope.

### Statistical analysis

Data analysis was performed by GraphPad Prism 9.5.1. The results were expressed as mean ± standard deviation. Differences between groups were analysed by the independent samples t-test and two-way ANOVA. *P *< 0.05 was considered significant.

## Results

### MC-B facilitated jawbone reconstruction in rats

**Figure 1.**
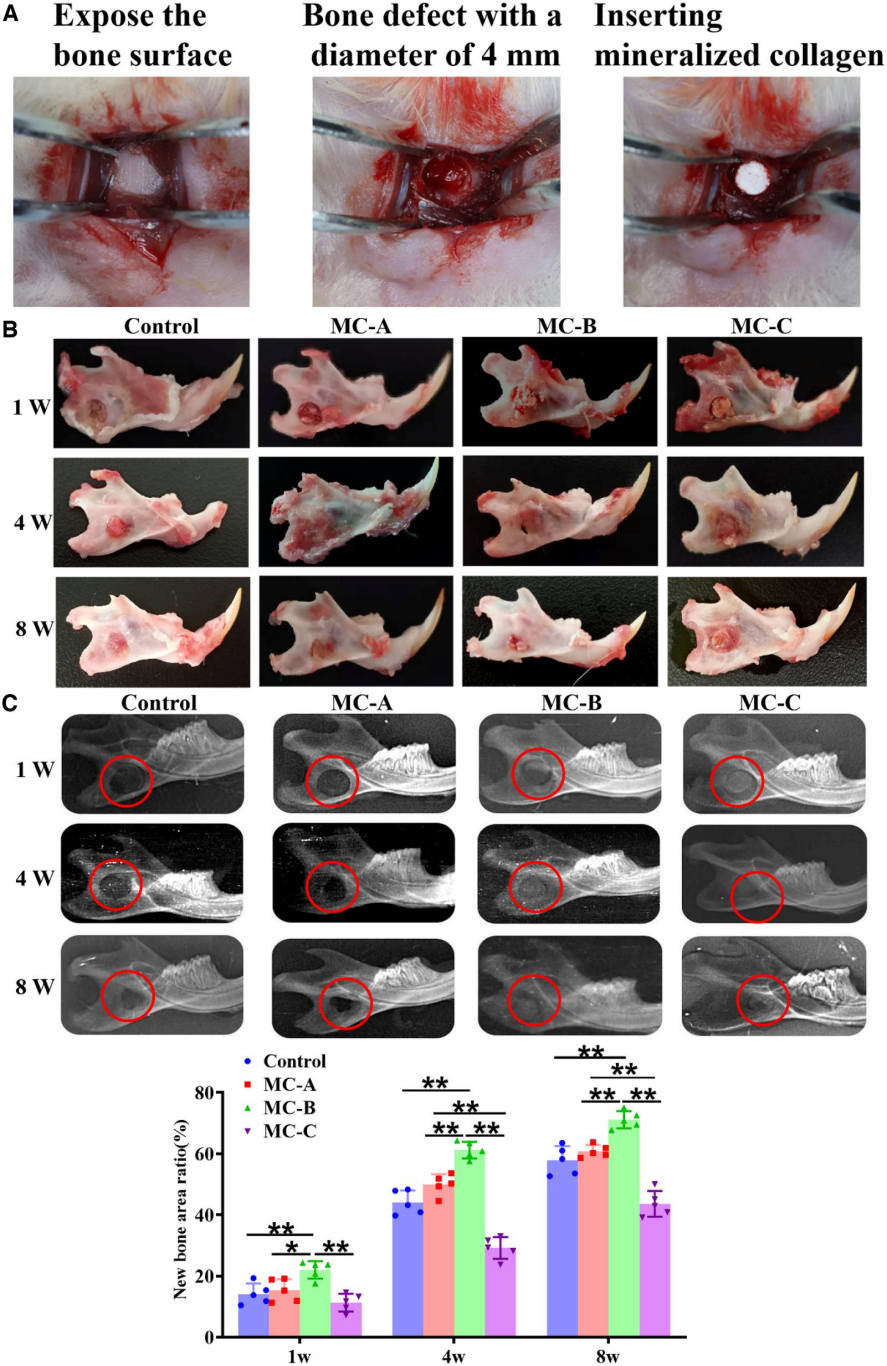

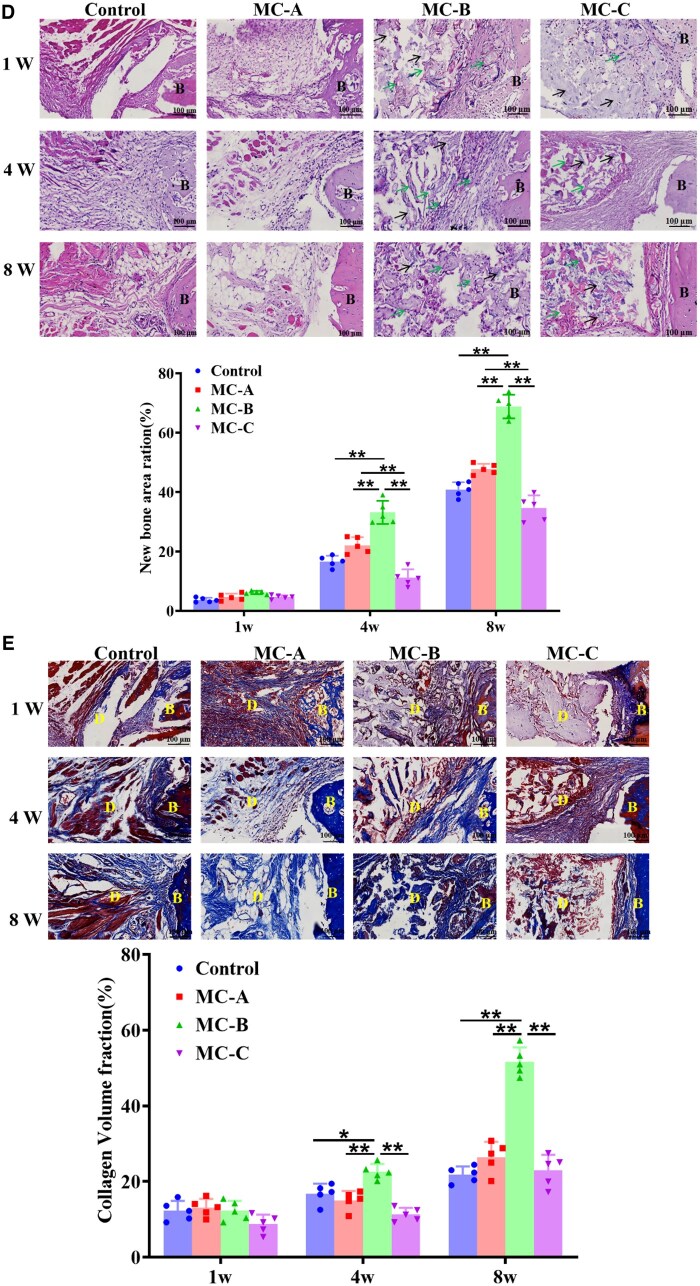
MC-B facilitated jawbone reconstruction in rats. (**A**) The preparation process of rat mandibular defect model. (**B**) General observation of rat mandibular specimens at 1 week, 4 weeks and 8 weeks after operation. (**C**) Imaging analysis of bone regeneration in the area of mandibular defects and semi-quantitative evaluation of new bone area in each group. (**D)** HE staining was used to observe tissue regeneration in the mandibular defects and semi-quantitative analysis on the area of nascent bone matrix in each group (scale: 100 μm, B: bone). The black arrow indicated MC scaffold, and the green arrow indicated new bone matrix. (**E**) Collagen neogenesis in bone defect areas was observed by Masson staining and semi-quantitative analysis was performed on the volume of neocollagenous tissue (scale: 100 μm, B: Bone, D: Defct).* P < 0.05; ** P < 0.01.

In previous studies, we found that MC microporous structure can regulate macrophage phenotype. The MC scaffold with a micropore structure of about 85 μm and 70% porosity (MC-B group) promotes the proliferation, spreading and osteogenic differentiation of MC3T3-E1 cells by inducing macrophage M2 polarization [14]. To determine the potential of MC with different apertures in the treatment of jawbone defects, we implanted MC in the rat mandible after preparing a 4 mm diameter defect (Figure 1A). At 1 week, 4 weeks and 8 weeks after operation, the mandible was taken, and the general observation results are shown in Figure 1B. The control group was filled with soft tissue within the defect one week after surgery. In addition, the MC-A group had the most obvious degradation of the material and the tightest bond with the soft tissue. At 8 weeks postoperatively, the extent of bone defects in the control, MC-A and MC-B groups was reduced compared with that at 4 weeks postoperatively. The material degradation was not obvious in the MC-C group. The MC-B group had the most significant bone repair as the area of bone defect had been filled with new bone. X-rays showed that there was a boundary between the material and normal bone tissue in all three groups at 1 week postoperatively. At 8 weeks after surgery, the bone repair effect was more obvious in the four groups than at 4 weeks. The MC-B group had the most significant high-density shadowing compared to the other groups. Semi-quantitative analysis of new bone area showed that the amount of new bone formation in MC-B group was higher than that in other groups at 4 weeks and 8 weeks. While MC-A had no significant difference to control group, MC-C had the worst bone repair (Figure 1C). HE staining showed that inflammatory cell infiltration was observed in all groups at 1 week after operation. The new bone trabecula in MC-B group was the most obvious at 4 weeks. At 8 weeks after surgery, the bone defects in the control and MC-A groups were mainly filled with fibrous connective tissue. Most of the material in the MC-B group had degraded, and the new bone matrix was significantly more abundant than other groups. The material in the MC-C group was clearly visible and surrounded by fibrous tissue. The semiquantitative analysis of new bone matrix showed that there was no meaningful difference in the amount of new bone formation between the groups at 1 week after surgery. The new bone matrix area was not significantly different in the control and MC-A groups, and was highest in the MC-B group and lowest in the MC-C group at 4 and 8 weeks (Figure 1D). Masson staining and semi-quantitative analysis are shown in Figure 1E. A small amount of blue-stained collagen fibres were observed of each group, 1 week after surgery. At 4 weeks, the MC-B displayed a large quantity of blue stained neoplastic bone matrix, while the other three groups were mainly collagen fibres. At 8 weeks, the blue-stained area of the bone defect site increased and red-stained mature bone tissue was found in the MC-B group. The control group was predominantly muscle fibres and soft tissue, the MC-A group was mainly fibrous connective tissue, and the MC-C group had a small amount of new bone matrix. Semiquantitative analysis revealed that the collagen volume fraction increased gradually with time in all groups, and was markedly higher in the MC-B group than in the other groups at 4 and 8 weeks.

### MC-B facilitating macrophage M2 polarization in bone defect areas

**Figure 2.**
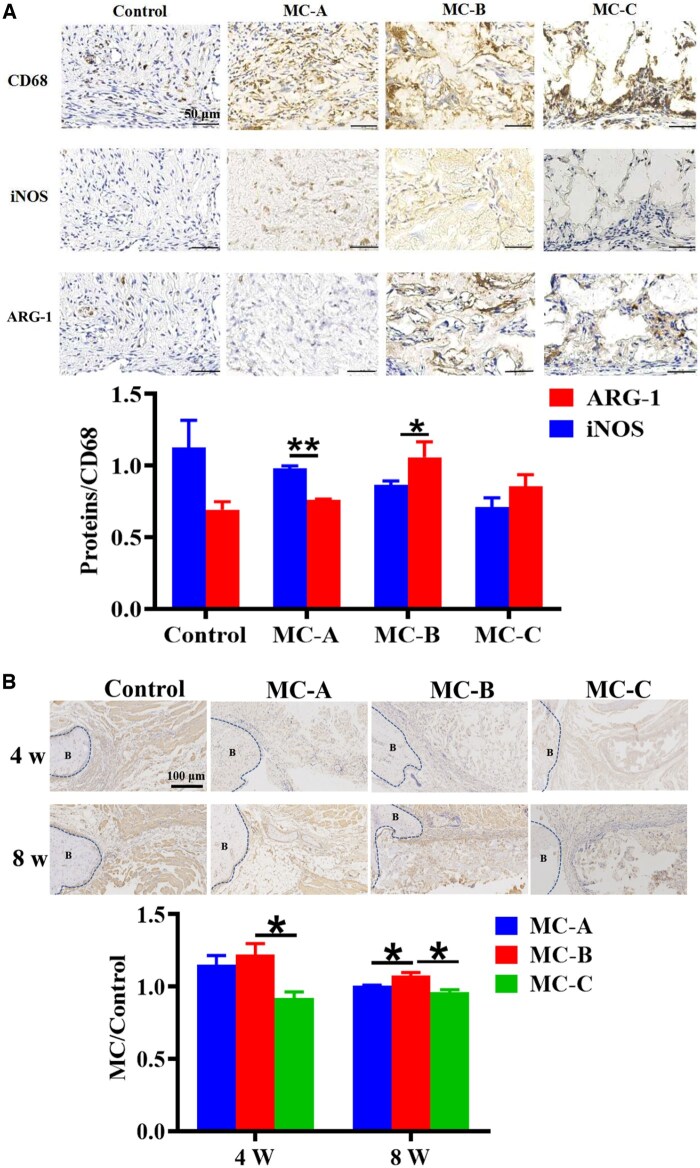
MC-B promoted bone healing by facilitating macrophage M2 polarization in bone defect areas. (**A**) The infiltration and polarization of macrophages in the mandibular defect area of rats after 1 week of MC implantation (scale: 50 μm). (**B**) The expression of OCN in mandibular defect area of rats 4 weeks and 8 weeks after MC implantation (scale: 50 μm).

To determine the function of macrophage polarization in bone injury repair, immunohistochemical staining was performed on rat mandibular specimens. The expression of CD68 (macrophage marker), iNOS and ARG-1 in the bone defect area was observed 1 week after surgery. The expression of OCN was observed at 4 weeks and 8 weeks. Figure 2A showed that the expression of both iNOS and ARG-1 was insignificant in control group and MC-C group; iNOS positive cells were significantly higher than ARG-1 positive cells in MC-A group; ARG-1 positive cells were higher than iNOS positive cells in MC-B group. Figure 2B found that the number of OCN positive cells in MC-B group was significantly higher.

### MC-B induced high expression of NCL in macrophages

However, it was not clear the mechanism by which MC-B regulated macrophage M2 polarization. Therefore, we used transcriptome sequencing to screen DEGs in macrophages regulated by MC-B MC. It was discovered that compared with the MC-A group, 2645 genes were up-regulated and 2695 genes were down-regulated in the MC-B group. Compared with MC-C, there were 737 up-regulated genes and 947 down-regulated genes in MC-B group (Figure 3A and B). The venn diagram showed that 458 genes were up-regulated in the MC-B group compared to the other two groups (Figure 3C and D). The heat map clustering analysis showed that the NCL gene was most significantly up-regulated in the MC-B group (Figure 3E). The expression of NCL was verified by qRT-PCR, and it was found that NCL expression was highest in the MC-B group (Figure 3F). GO analysis indicated that macrophages in the MC-B group were mainly involved in chromosomal region, nuclear chromosome and DNA repair (Figure 3G). KEGG enrichment suggested that macrophages in the MC-B group were mainly involved in mismatch repair, DNA replication, cell cycle, homologous recombination spliceosome, fanconi anemia pathway (Figure 3H). The results of immunohistochemistry showed that MC-B had the highest number of NCL positive cells (Figure 3I).

**Figure 3.**
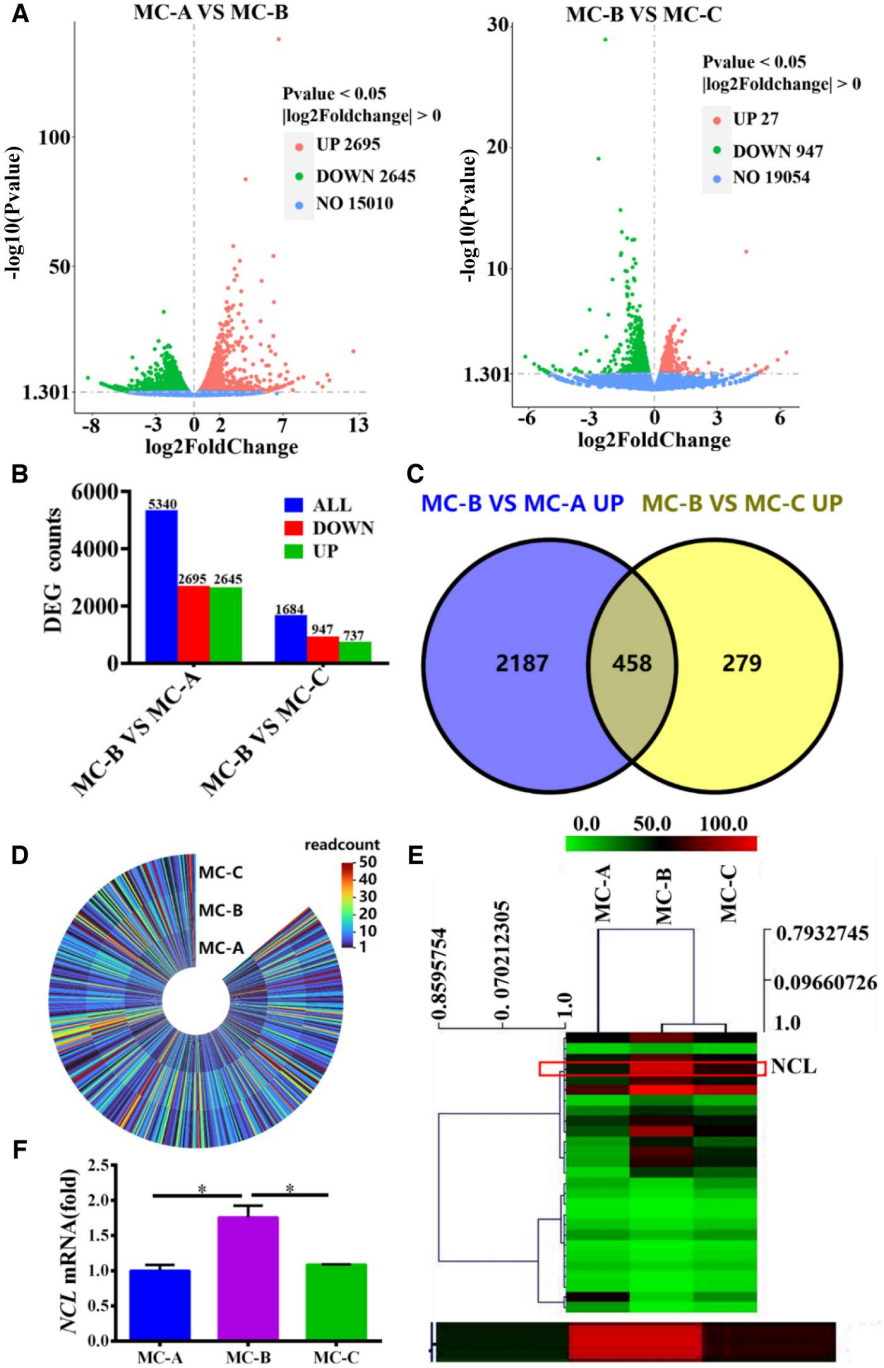
NCL was highly expressed in MC-B group. (**A**) Volcano map of DEGs. (**B**) The number of DEGs. (**C**) Venn diagram of highly expressed genes in MC-B group. (**D**) Heatmap of highly expressed genes in MC-B group. (**E**) Heatmap for cluster analysis of DEGs. (**F**) The expression of NCL was verified by qRT-PCR. (**G**) GO analysis of DEGs in each group. (**H**) KEGG pathway enrichment analysis of DEGs in each group. (**I**) Immunohistochemical staining was used to observe the expression of NCL positive cells in rat mandibular bone defect area (scale: 100 μm).*P < 0.05.

**Figure 3.**
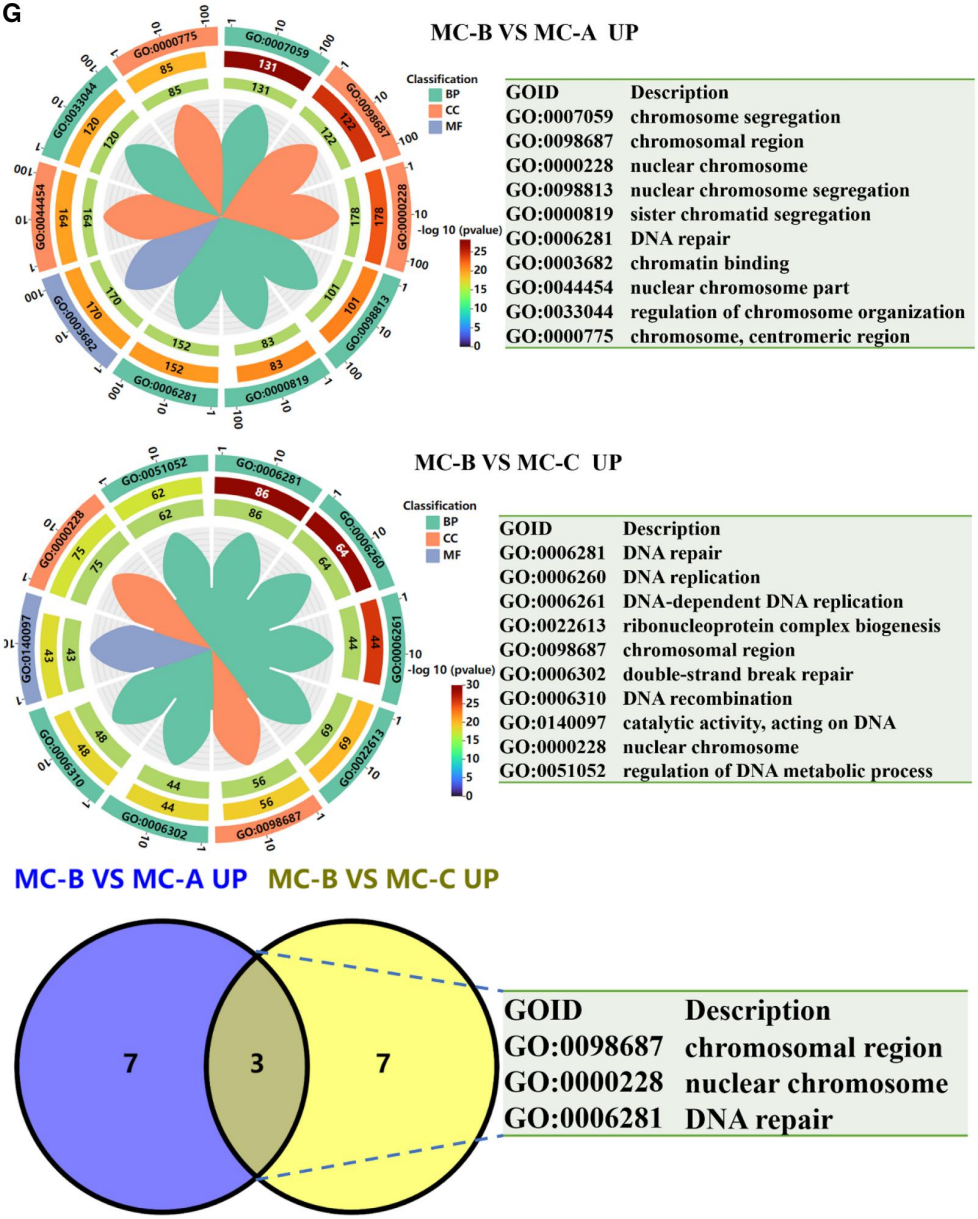
(Continued)

**Figure 3.**
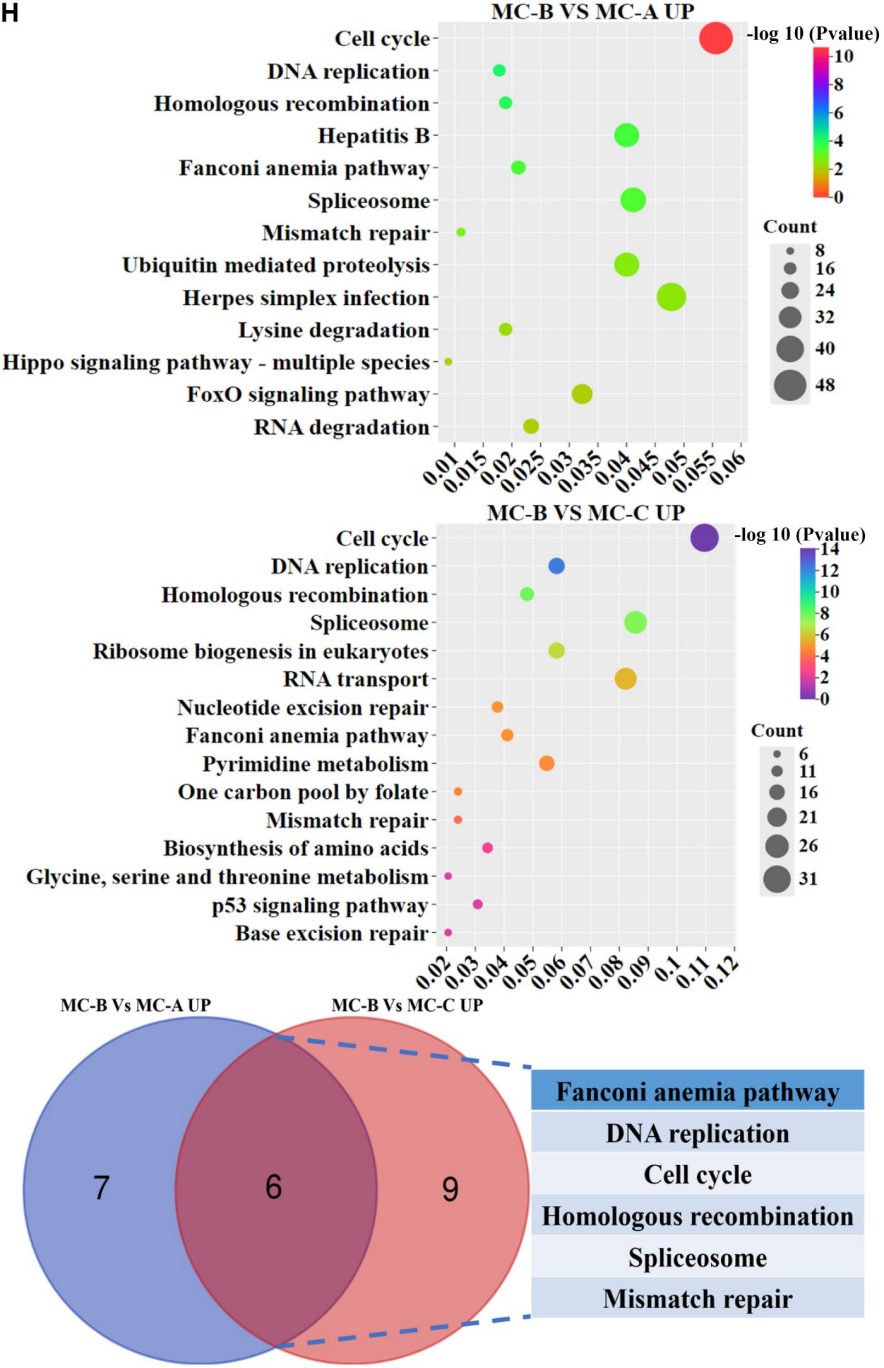
(Continued)

**Figure 3.**
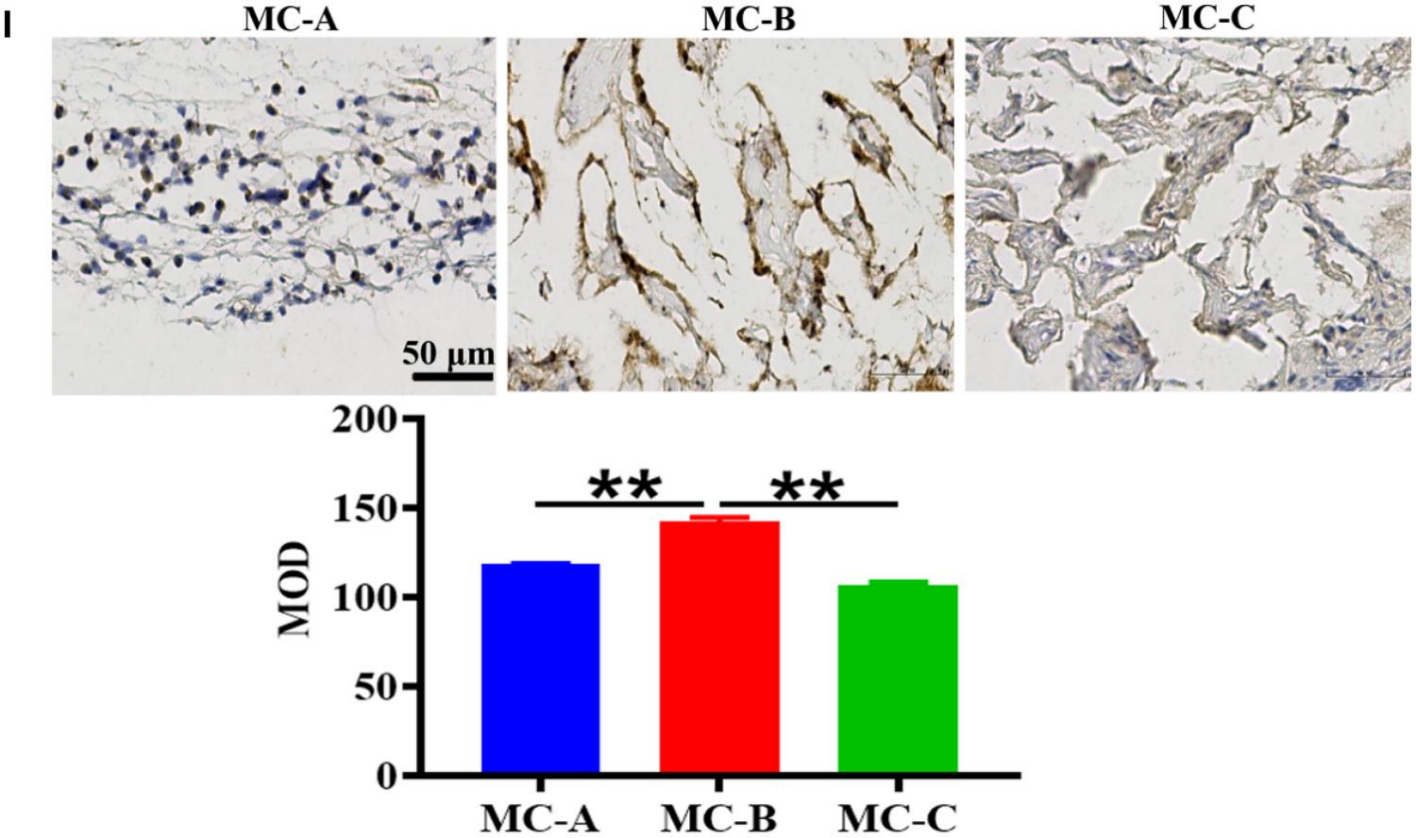
(Continued)

### Inhibition of NCL impeded macrophage M2 polarization

**Figure 4.**
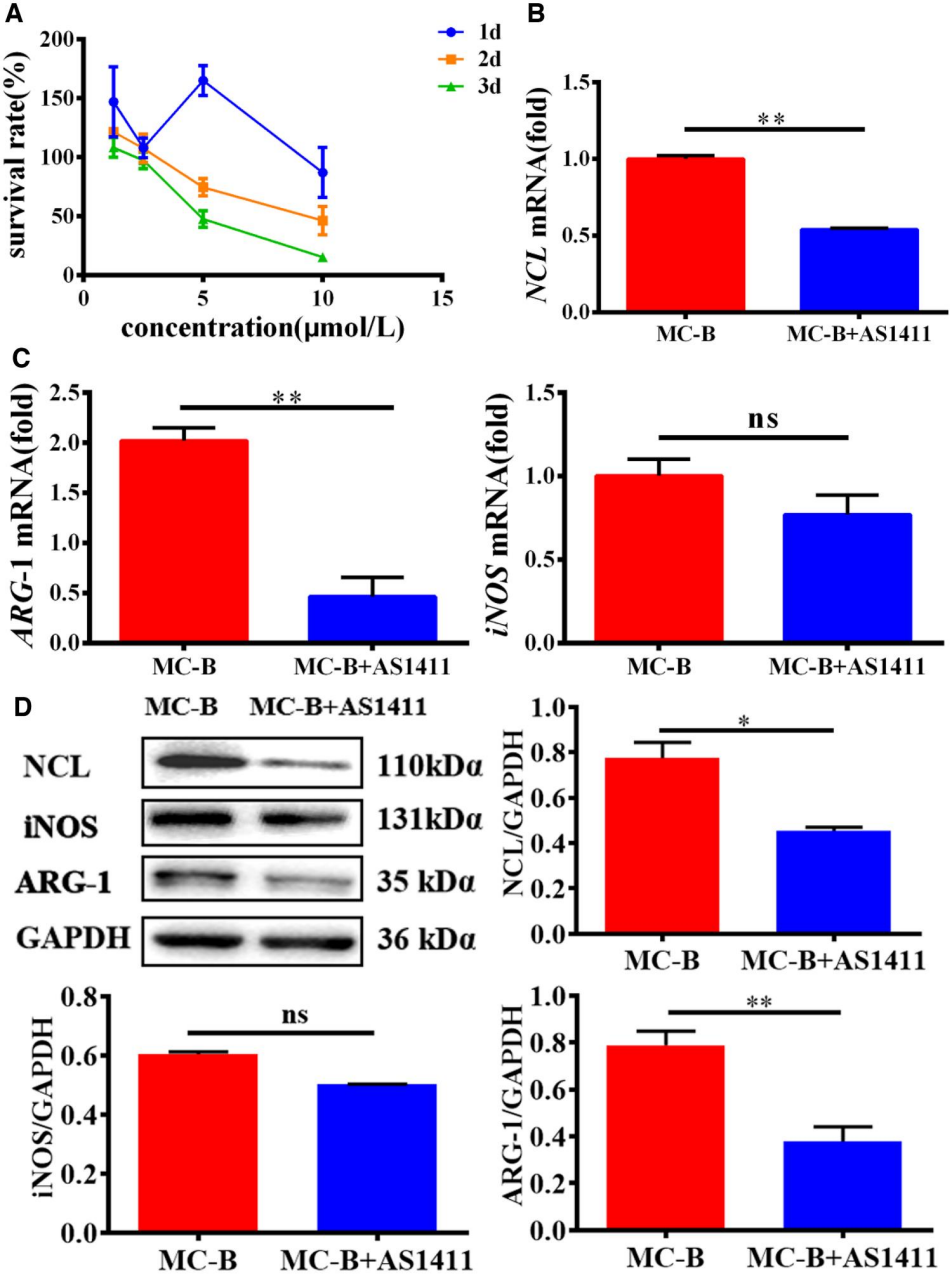
Inhibition of NCL impeded macrophage M2 polarization. (**A**) The effect of AS1411 on macrophage viability was detected by CCK-8. (**B**) The inhibitory effect of AS1411 on NCL in macrophages was detected by qRT-PCR. (**C**) The effects of AS1411 on ARG-1 and iNOS mRNA in macrophages were detected by qRT-PCR. (**D**) The effect of AS1411 on NCL, ARG-1, iNOS protein expression in macrophages was detected by Western blot.

Next, we explored whether NCL effected macrophage polarization. NCL inhibitor AS1411 was included in the culture medium at different concentrations, and the survival rate of macrophages was inhibited with the increase of AS1411 concentration. IC50 was 5 μmol/L (Figure 4A). After culturing macrophages on MC-B MC surface with AS1411 at a concentration of 5 μmol/l, NCL expression was detected by qRT-PCR and was found to be significantly inhibited (Figure 4B). Moreover, the mRNA expression of ARG-1 was significantly reduced, while the iNOS was not obviously different compared with the MC-B group (Figure 4C). As shown in Figure 4D, AS1411 inhibited the expression of NCL and ARG-1 protein, but had no significant effect on iNOS.

### The NCL promoted macrophage polarization to M2

**Figure 5.**
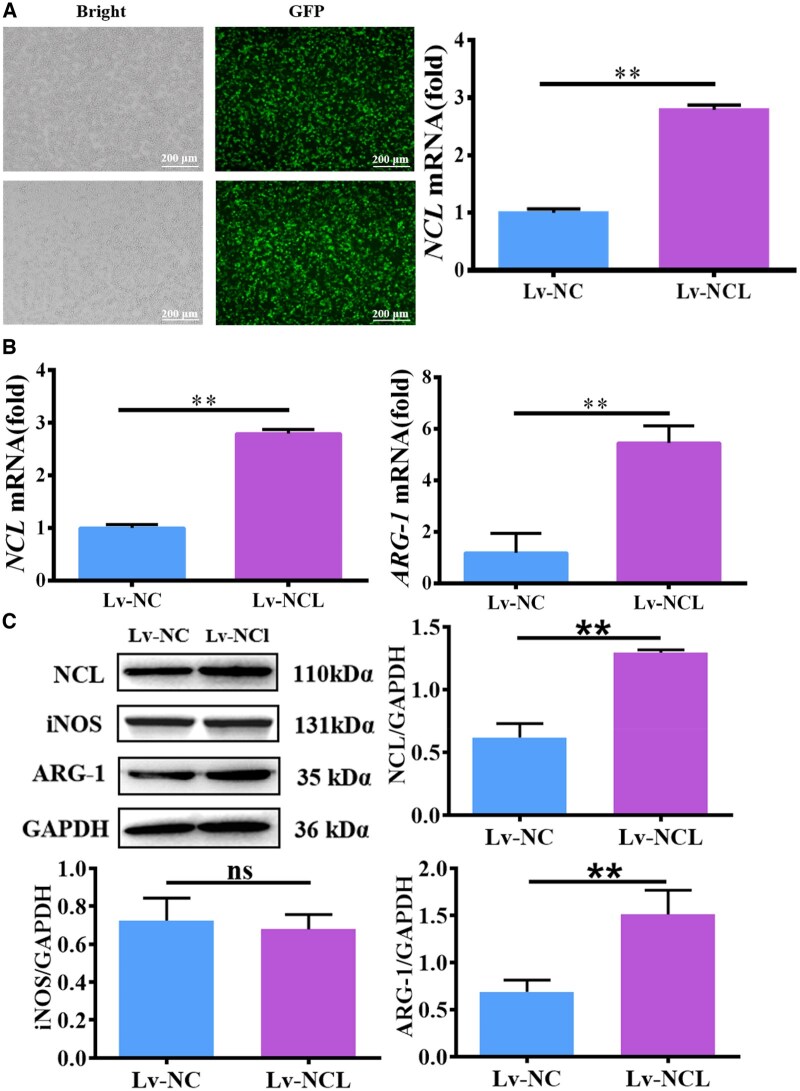
The NCL promoted M2 macrophage polarization. (**A**) Green fluorescent protein (scale bar: 200 μm) and NCL gene expression after lentivirus transfection in macrophages. (**B**) The effect of NCL on iNOS and ARG-1 gene expression. (**C**) The effect of NCL on iNOS and ARG-1 protein expression.

Subsequently, NCL was overexpressed in macrophages. RAW264.7 cells were transfected with lentivirus overexpressing NCL, and strong green fluorescence was observed. The qRT-PCR analysis confirmed that the mRNA level of NCL in macrophages was significantly increased (Figure 5A). RAW264.7 cells transfected with lentivirus were seeded onto the surface of MC-B material and cultured for 3 days. Total RNA and protein were extracted to detect the expression of ARG-1 and iNOS. The findings revealed that both gene and protein expression of ARG-1 were increased in the NCL overexpression group, whereas there was no significant difference in the iNOS compared with the negative control group (Figure 5B and C).

## Discussion

Bone regeneration is a complex and precisely regulated process involving many molecular, cellular, biochemical and mechanical factors. Therefore, highly active bone substitute materials are required to be prepared into porous bone tissue engineering scaffolds with appropriate pore size and degradability to induce bone regeneration. The physical and chemical properties of bone repair scaffold materials can affect the biological behavior and function of cells [15–17]. It is one of the critical factors for the successful application of biomaterials that the surface of the material interacts with the cells. A growing number of researches have shown that microporous structures on the surface of biomaterials can effectively regulate cell adhesion, extension, proliferation and differentiation [18]. Pore size is a critical feature of tissue engineering scaffolds. Microporous structure provides a good space for cell growth, migration and interaction. Numerous studies had demonstrated that the microporous structure can influence the biological behaviour of MSCs, and that porous materials were more likely to promote the osteogenic differentiation of MSCs than non-porous materials [19–21]. Small pore size can promote cell adhesion, but it is easy to form hypoxic environment. The large pore size can achieve efficient penetration of nutrients in the scaffold, but it tends to decrease the mechanical and compressive strength of the scaffold, which is not conducive to maintaining the osteogenic space. Moreover, the influence on osteogenic function of microporous structure size depended on the material [22–24]. Chen *et al*. found that titanium implants with a pore size of 500 μm and a porosity of 60% showed the best cell proliferation and osteogenic differentiation *in vitro* and bone growth *in vivo* [25]. As an artificial bone material prepared by biomimetic mineralization technology, the microporous structure of MC played a crucial role in promoting bone tissue regeneration and repair. The porous structure of MC not only allowed cells, blood vessels and new bone tissues to grow into the pores of the material, but also promoted the tight binding of these tissues to the material. The suitable microporous structure of MC and the effect of changes in microporous structure on bone regeneration and maintenance of osteogenic space need to be explored. In this research, a rat mandibular full penetration type critical bone defect model was prepared, and MC with different microporous structures was implanted into the defect area to evaluate its osteogenic ability. Masson staining is mainly used for collagen staining. The results of collagen staining in bone tissue were related to the maturity of collagen, and collagen blue staining in new bone and early immature bone tissue. With the maturation of bone, collagen staining gradually changed from blue to red. Hematoxylin-eosin staining was used to observe inflammatory cell infiltration and new bone formation in bone defect area. The outcomes demonstrated that the deposition of new bone and the degradation of the material in the MC-B group (85 μm) were synchronized and the best growth rate of new bone was achieved (Figure 1).

Previous studies on bone biomaterials mainly focused on the influence of materials over the biological behavior of osteoblasts. However, it is not enough to explain the phenomenon in bone repair, and the explanation of the mechanism is still superficial. The host immune response is the first to occur when biomaterials are implanted in the body, with macrophages playing a key role. Being important effector cells in the body’s intrinsic immunity, macrophages regulate the immune response at various stages of bone regeneration in the bone microenvironment by polarizing into pro-inflammatory M1-type and inhibitory M2-type in response to environmental signals [26–28]. The microporous structure of MC modulated macrophage polarization, which in turn affected the process of osteogenic differentiation. M2 macrophage markers (ARG-1 and CD206) were significantly upregulated after co-culture of medium pore size MC and macrophages [14]. However, it was not clear the mechanism by which MC-B regulated macrophages during bone repair.

Transcriptome is the sum of all transcripts and their quantities of cells at specific developmental stages or physiological conditions. Transcriptomics can reveal the expression levels and patterns of genes in specific physiological or pathological conditions, so as to deeply understand the role of genes in organisms and their regulatory mechanisms [29]. In this research, transcriptome sequencing was used to screen DEGs in three groups of macrophages regulated by MC, and the DEGs were verified by qRT-PCR. It was found that macrophages co-cultured with MC-B highly expressed NCL and activated the fanconi anemia pathway. Moreover, MC-B promoted the expression of NCL in the jaw defect area of rats (Figure 3). NCL is the most important protein in the nucleolus of eukaryotic cells. It not only participates in the biosynthesis of ribosomes, but also participates in cell proliferation, growth, chromatin replication and nucleolus formation. It is mainly expressed on the surface of proliferating cells, and also expressed on the surface of macrophage cell membrane [30–32]. Previously, the role of NCL in promoting tumour proliferation was mainly investigated, and the relationship between it and macrophage polarization was less researched at present. Tang *et al*. found that NCL deficiency reduced M2 macrophage polarization thereby impairing cardiac function during recovery from myocardial infarction [33]. We further explored the role of NCL that promoted macrophage M2 polarization in MC-B to provide new targets for precise regulation of macrophage polarization.

**Figure 6.**
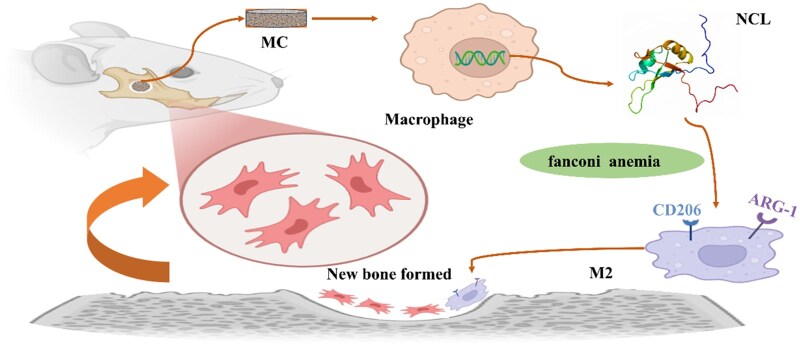
The mechanism of MC regulating macrophage polarization affecting bone regeneration.

AS1411 is a nucleic acid aptamer of NCL, which can specifically bind to NCL and inhibit NCL function [34]. AS1411 was used to inhibit NCL in macrophages, and ARG-1 was suppressed (Figure 4). In addition, lentiviral vectors for the NCL gene were selected in this study, and a stable transfected cell line was constructed by integrating the exogenous NCL gene into cells through lentiviral infection of macrophages. And the expression of ARG-1 was upregulated after overexpression of NCL in macrophages (Figure 5). Therefore, NCL was involved in the process of MC-B regulating the polarization of macrophages to M2 type (Figure 6).

## Conclusion

In conclusion, based on the research concept of bone immune microenvironment regulation of bone regeneration, the present study was carried out to investigate the effect of different microporous structures of MC modulating macrophage polarization on bone regeneration. It was revealed that MC with a pore size of about 84 μm induced macrophage to M2 polarization by promoting the expression of NCL to facilitate the repair of jaw defects, and the fanconi anemia pathway was involved in this process. However, whether NCL can be combined with MC, metal stents, hydrogels, 3D-printed materials and other bone repair materials to endow them with effective targeting capabilities for bone repair remains to be further explored. Overall, this investigation provided a theoretical basis for the development of bone replacement materials with targeted regulation of the bone immune microenvironment, and provided ideas for establishing new strategies for bone repair.

## Data Availability

Data will be made available on request.
